# Effectiveness and safety of photobiomodulation therapy in diabetic peripheral neuropathy: Protocol for a systematic review and meta-analysis

**DOI:** 10.1371/journal.pone.0308537

**Published:** 2024-08-26

**Authors:** Xuechun Fan, Guanchi Yan, Jingsi Cao, Yunyun Zhao, Ying Wang, Xiuge Wang, Jia Mi

**Affiliations:** 1 College of Traditional Chinese Medicine, Changchun University of Chinese Medicine, Changchun, China; 2 Department of Endocrinology, First Affiliated Hospital to Changchun University of Chinese Medicine, Changchun, China; Massachusetts General Hospital, UNITED STATES OF AMERICA

## Abstract

**Introduction:**

Diabetic peripheral neuropathy (DPN), a widely prevalent complication in patients with type 2 diabetes, exerts a significant influence on patients’ overall health and financial circumstances. Photobiomodulation therapy is one of the means of physical therapy for DPN. Although preliminary findings suggest the efficacy of photobiomodulation therapy in alleviating peripheral neuropathy, the existing literature lacks substantial evidence regarding its safety and effectiveness specifically in the context of diabetes-related peripheral neuropathy. Therefore, we plan to arrive at more distinct findings through systematic evaluation and meta-analysis.

**Methods:**

We will conduct a comprehensive search for studies published from the beginning until October 1, 2023, using various databases including Web of Science, Embase, Cochrane Library, PubMed, AMED, Wanfang database, VIP database, China National Knowledge Infrastructure, and the Chinese Biomedical Literature database. Simultaneously, we will also search for the WHO International Clinical Trial Registration Platform, China Clinical Trial Registration Platform, and Clinical Trials.gov. Gray literature will be retrieved using Google Scholar and opengrey.edu. Only randomized controlled trials in Chinese and English were included, with no restrictions on publication status. The primary outcomes will include change of symptom scores, change of nerve conduction velocity. Additional outcomes will encompass quality of life, change in pain, blood glucose levels after fasting and 2 hours after eating, levels of glycosylated hemoglobin, and any adverse events associated with photobiomodulation therapy. Reman V.5.4 and R language will be used for the meta-analysis. Assessment of potential bias will be conducted through Cochrane risk of bias 2 tool (RoB 2.0) and Physiotherapy Evidence Database (PEDro) scale. Registration: PROSPERO (registration number: CRD42023466586).

**Discussion:**

This meta-analysis aims to assess the efficacy and safety of photobiomodulation therapy as a potential treatment for diabetic peripheral neuropathy (DPN), and providing a straightforward and convenient therapeutic for patients. Additionally, it expands the range of treatment alternatives available to healthcare professionals managing DPN.

## 1. Introduction

The incidence of diabetes mellitus (DM) is steadily rising as a result of significant changes in lifestyles and dietary habits. Based on estimation from the International Diabetes Federation, it is anticipated that around 33% of the population will be impacted by diabetes by the year 2050, with nearly half of them developing neuropathy if glycemic control is not effectively achieved [1, 2].

DPN has become the prevailing type of neuropathy globally, accounting for the highest occurrence rate [3]. DPN has the potential to induce a variety of negative outcomes, such as falls and feelings of depression [4]. Moreover, DPN additionally raises the probability of ulceration, infection, and amputation, thereby exacerbating the financial burden in patients with T2DM [5, 6].

DPN treatment currently involves managing glycemic levels, providing relief for pain symptoms, and improving the function of peripheral nerves [7]. Nevertheless, the management of blood glucose levels has limited impact on individuals diagnosed with type 2 DPN [8]. In addition, although many drugs are used to alleviate pain such as antidepressants and anticonvulsants [9], these drugs do not provide significant pain relief and can lead to drug dependence and related serious adverse effects [10, 11].

Non-pharmacological therapies have the characteristics of good safety and compliance. And they are easily accepted by patients. With the advancement of technology, physical therapies, especially phototherapies, have become an important component of non-pharmacological therapies. Studies have indicated that low level laser therapy (LLLT) treats DPN by alleviating clinical symptoms and improving neurological function [12, 13]. Photobiomodulation therapy can improve neuropathic pain in diabetic peripheral neuropathy [14]. Low-intensity laser therapy (LILT) plays an active role in improving the nerve conduction velocity of patients with diabetes peripheral neuropathy [15]. In addition, multiple types of photobiomodulation therapy are used to the treatment of DPN. Hence, photobiomodulation therapy shows great potential as one of the approach for the clinical treatment of DPN. However, the mechanisms of action of phototherapies remain unclear so far. The mechanism of action of phototherapies can be due to increase mitochondrial ATP production [16], decrease in serum Neuron Specific Enolase (NSE) levels [17] or decrease inflammation [18] et al. Currently, there are only a few systematic reviews of single photobiomodulation therapy. These systematic reviews mainly focuse on research in neuropathic pain [19], plantar pressure distribution [14] and plantar tactile sensitivity [20].

However, there is currently no systematic review or meta-analysis of photobiomodulation therapy to synthesis these evidences. Hence, the objective of the study is to assess the effectiveness and safety of photobiomodulation therapy in the treatment of individuals suffering from diabetic peripheral neuropathy. We anticipate that this research will provide valuable findings for the future treatment of photobiomodulation therapy in individuals with DPN.

## 2. Materials and methods

### 2.1. Registration of the study

The design of the protocol conforms to the guidelines specified in the Preferred Reporting Items for Systematic Reviews and Meta-analyses Protocols 2015 [21]. The study has been registered with the International Prospective Register of Systematic Reviews (PROSPERO) (registration number CRD42023466586).

### 2.2. Inclusion criteria

#### 2.2.1. Types of study

We will include randomized controlled trials (RCTs) of photobiomodulation therapy for patients with diabetes peripheral neuropathy, while quasi-RCTs will not be specifically excluded. Non-randomized clinical trials, duplicated publications, conference records, reviews, meta-analyses, newspapers, guides, letters, other documents, and studies without full text will be omitted. Publication will be limited to English and Chinese, with no limitations on the date of publication.

#### 2.2.2. Classification of participants

Our study will include adults (over 18 years of age) with any form of diabetes who have been diagnosed with DPN. There will be no restrictions on the age, gender, ethnic background, or nationality of the participants who are enrolled. Exclusion will occur for studies involving pregnant or other types of peripheral neuropathy.

#### 2.2.3 Type of outcome

The primary outcomes will include change of symptom scores (Neurological Impairment Scale [NIS] or similar scales), change of nerve conduction velocity. In addition to photobiomodulation therapy-related adverse events, the secondary outcomes will encompass quality of life (Health-Related Quality of Life scale or related scores), Change in pain (Visual Analogue Scale, Numeric Rating Scale and so on), fasting blood glucose, blood glucose 2 hours after eating, and glycosylated hemoglobin.

#### 2.2.4 Type of interventions

There will be no restrictions on the type or frequency of light, the wavelength or equipment that produces light. The light can come from LEDs, low level lasers, or lamps. We will include randomized controlled trials that meet any of the following intervention comparisons: photobiomodulation therapy and pseudo photobiomodulation therapy, photobiomodulation therapy and placebo, photobiomodulation therapy and non-specific treatment other than conventional treatment, or photobiomodulation therapy plus another treatment and another treatment in addition to conventional treatment.

### 2.3. Search strategy

The search strategy will be based on the guidelines provided by the Cochrane Handbook for Systematic Reviews of Interventions [22].

### 2.4. Electronic searches

The study will encompass papers published from the inception until October 1, 2023, employing terminology such as photobiomodulation therapy, diabetic peripheral neuropathy, and randomized controlled trial. Web of Science, Embase, Cochrane Library, PubMed, AMED, Wanfang database, VIP database, China National Knowledge Infrastructure, and the Chinese Biomedical Literature database will all be queried during the search. Table 1 displays the detailed search method for PubMed.

**Table 1:** Search strategy in PubMed.

Number	Search items
#1	Photobiomodulation Therapy [mh]
#2	Phototherapy [tiab]
#3	Phototherapies [tiab]
#4	Photoradiation therapy [tiab]
#5	Therapy, Photoradiation [tiab]
#6	Photoradiation Therapies [tiab]
#7	Therapies, Photoradiation [tiab]
#8	Light Therapy [tiab]
#9	Light Therapies [tiab]
#10	Therapies, Light [tiab]
#11	Therapy, Light [tiab]
#12	Light emitting diode therapy [tiab]
#13	Low level light therapy [tiab]
#14	Light [tiab]
#15	Photobiomodulation [tiab]
#16	Infrared [tiab]
#17	Laser Phototherapy [tiab]
#18	Low level laser therapy [tiab]
#19	Cold laser [tiab]
#20	OR/#1—#19
#21	Diabetic Neuropathies [mh]
#22	Diabetic Neuropathy [tiab]
#23	Diabetic Autonomic Neuropathy [tiab]
#24	Painful Diabetic Neuropathy [tiab]
#25	OR/#21—#24
#26	Randomized controlled trial [mh]
#27	Controlled clinical trial [tw]
#28	Clinical Trial [tw]
#29	Randomised [tw]
#30	Randomly [tw]
#31	Trial [tw]
#32	Clinical Study [tw]
#33	Random allocation [tw]
#34	OR/#26—#33
#35	#20 AND #25 AND #34

### 2.5. Other search strategy

We will examine additional ongoing and unpublished studies listed in the WHO International Clinical Trial Registration Platform(https://trialsearch.who.int/), China Clinical Trial Registration Platform(http://www.chictr.org.cn/), and Clinical Trials.gov (https://clinicaltrials.gov/). Additionally, the relevant systematic reviews’ reference lists will be thoroughly examined by manual means. The retrieval of gray literature conducted through the utilization of Google Scholar and opengrey.edu.

### 2.6. Study selection

**Fig 1:**
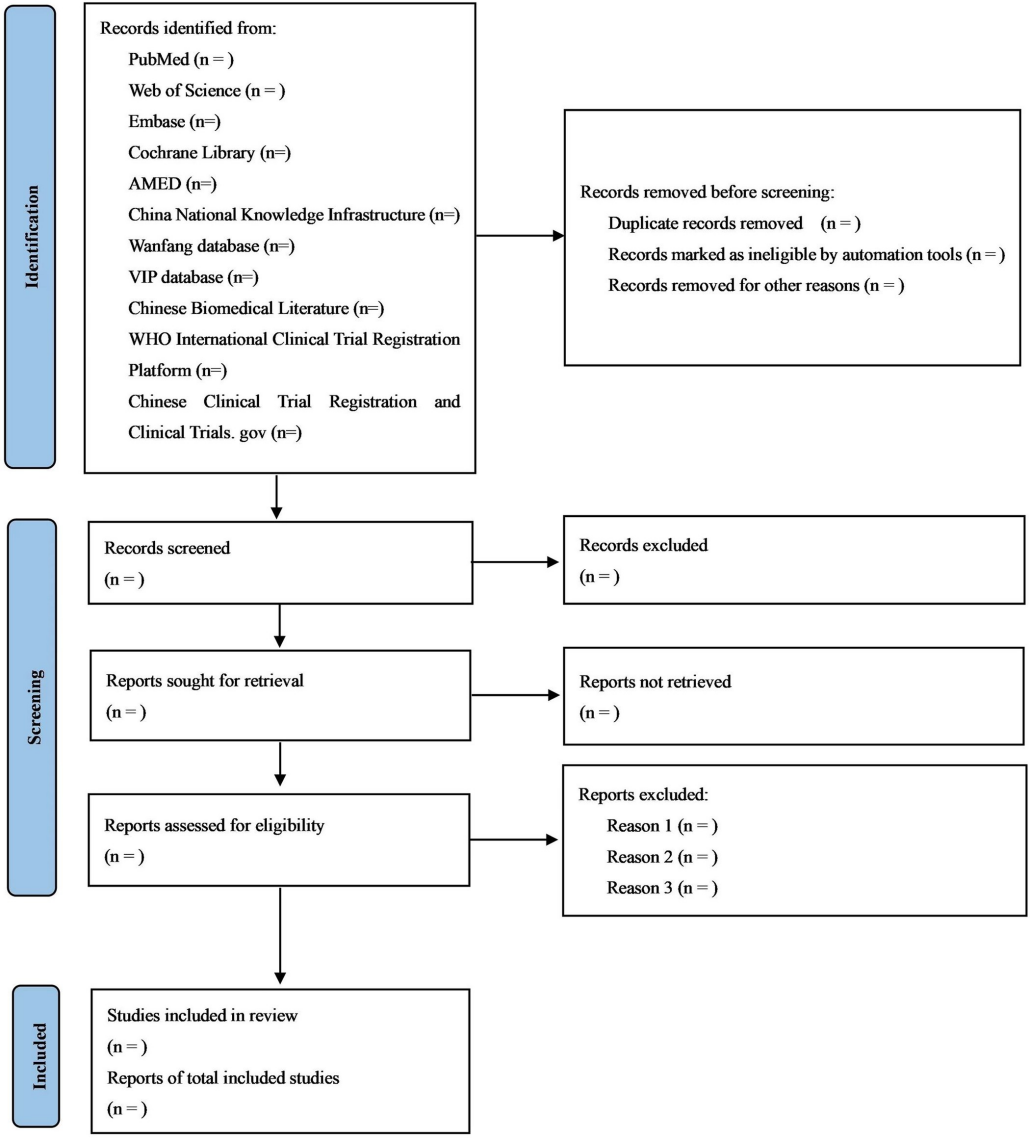
Screening flowchart.

Two researchers will conduct separate evaluations of the studies. The outcomes will be examined and any discrepancies will be discussed. During the course of the research period, any disagreements will be effectively solved by engaging in discussions or negotiations with a third researcher. Fig 1 displays a flowchart depicting the screening process, which is derived from the PRISMA flow diagram 2020 [23].

### 2.7. Data collection and analysis

After reading the complete text of the chosen papers, two researchers will independently extract the information:

1. The papers contain essential details (the name of primary author, the year of publication, country, language, and race).
2. Key characteristics of the patients (sample size, inclusion criteria, effectiveness criteria, Diabetes duration/DPN duration, and demographic baseline).
3. The details regarding the intervention and control groups, including the duration of intervention, type of intervention, and frequency of intervention.
4. Methodological characteristics (blinding and hidden allocation sequence).
5. The results include primary outcomes (symptom score, nerve conduction velocity) and secondary outcomes (quality of life scores, 2-h postprandial blood glucose, fasting blood glucose, glycosylated hemoglobin, and any adverse events associated with photobiomodulation therapy).

All conflicts will be settled by participating in discourse or deliberation with a tertiary researcher.

### 2.8. Risk of bias assessment

The Cochrane Risk of Bias 2 (RoB 2) tool [22] and Physiotherapy Evidence Database (PEDro) scale (https://www.pedro.org.au/english/downloads/pedro-scale/) were employed, two researchers will individually evaluate the potential for bias. The evaluation and assessment of all the studies will be conducted based on participant blinding, allocation concealment, outcome assessment blinding, result data integrity, selective outcome reporting, along with potential biases. The assessment results will be classified as low, high, or uncertain risk.

#### 2.8.1. Missing data

In case the included studies are missing crucial information, we will reach out to the primary investigator to acquire the necessary details. If the necessary information cannot be obtained, we will exclude these studies.

#### 2.8.2. Evaluation of heterogeneity

To evaluate the heterogeneity of the studies incorporated, we will employ the *χ*^2^ test and *I*^2^ value [22]. When the *P* values in the *χ*^2^ test are greater than 0.05 or the *I*^2^ is less than 50%, it indicates that there is homogeneity among the studies. When the value of *P* is less than or equal to 0.05 and the value of *I*^2^ is greater than or equal to 50%, this finding suggests that there is variability or diversity among the studies. To analyze the heterogeneity, we will implement subgroup analysis and meta-regression to analyze the origin of variability.

#### 2.8.3. Evaluation of reporting biases

Funnel plots can be utilized to reveal biases of reports when the number of included studies exceeds ten.

#### 2.8.4. Data synthesis

RevMan V.5.4 software will be used for meta-analysis. The weighted mean difference of the 95% CI will be used for continuous data and the risk ratios of the 95% CI will be used for dichotomous data. In case there are less than or equal to 3 studies included, we will furnish a descriptive and qualitative overview.

#### 2.8.5. Subgroup analysis

Analysis of subgroups will be carried out, taking into account the following factors: Photobiomodulation therapy type, diabetes type, intervention duration, course of diabetes, and duration of DPN.

#### 2.8.6. Sensitivity analysis

To measure the resilience and dependability of the findings, a sensitivity analysis will be conducted. Studies with a significant bias will be excluded. Furthermore, the impact of chosen models will be taken into account. Step-wise rejection method is used for sensitivity analysis.

#### 2.8.7. Meta-regression analysis

The process of meta-regression will be carried out in a same manner to linear regression, where the study estimate will serve as the dependent variable and the study characteristics [24] will act as the independent variables. In addition, we will use R language for meta-regression analysis.

### 2.9. Summary of findings

The GRADE will be employed to import data from Review Manager 5.4, in order to generate a table presenting the summary of findings [25]. Two researchers will independently assess the quality of evidence. Every result will be categorized into one of four ratings: high, medium, low, or very low.

## 3. Discussion

Diabetes peripheral neuropathy (DPN) is the most common cause of neuropathy worldwide, and its incidence rate increases with the course of diabetes [3, 26]. DPN has a significant impact on the quality of life of patients [27]. Doctors and patients are searching for an effective and safe non-pharmacological therapy due to the drawbacks of limited efficacy, multiple adverse reactions, and high cost in traditional pharmacological therapy. Photobiomodulation therapy has been increasingly used to evaluate its effectiveness in DPN related researches, due to its advantages of simple operation and low cost [14, 28].

Previous studies were focused on systematic review and meta-analysis for a single type of photobiomodulation therapy, without an evaluation of all types of phototherapies. Hence, we couldn’t have a clear cognition of the efficacies and differences between different phototherapies. We believe that it is necessary to perform a systematic review and meta-analysis of the current of literature to assess the efficacy and safety of photobiomodulation therapy in diabetic peripheral neuropathy. We are believe that clinicians will find the valuable of this study. Furthermore, we anticipate that this meta-analysis will offer a straightforward and convenient therapy for treating individuals with DPN. At the same time, it also provides doctors with more choices in the treatment of diabetes peripheral neuropathy.

## 4. Advantages and constraints

This systematic review provides a thorough examination of the efficacy and safety of photobiomodulation therapy in treating patients with DPN. Due to the assessment of only RCTs, the inclusion criteria are stringent. To assessment the efficacy of photobiomodulation therapy, there will be no limitations on the type or frequency of light, the wavelength or equipment that produces light. The different types of photobiomodulation therapy will not have any restrictions, which may result in significant variation among studies.

## Supporting information

S1 Checklist PRISMA-P 2015 checklist. (DOCX)

S1 Table Search strategy. (DOCX)
