# Supplementary material for: Effectiveness and safety of photobiomodulation therapy in diabetic peripheral neuropathy: Protocol for a systematic review and meta-analysis
Source: PLoS One. 2024 Aug 26;19(8):e0308537. doi: 10.1371/journal.pone.0308537 (PMC11346721; doi:10.1371/journal.pone.0308537)
Supplement: S1 Checklist — (DOCX) [file pone.0308537.s001.docx]

**PRISMA-P 2015 Checklist**

# **This checklist has been adapted for use with protocol submissions to *Systematic Reviews* from Table 3 in Moher D et al**:**** Preferred reporting items for systematic review and meta-analysis protocols (PRISMA-P) 2015 statement. *Systematic Reviews* 2015 ****4****:1

| **Section/topic** | **#** | **Checklist item** | **Information reported** | | **Line number(s)** |
| --- | --- | --- | --- | --- | --- |
|  |  |  | **Yes** | **No** |  |
| **ADMINISTRATIVE INFORMATION** | | | | | |
| **Title** | | | | | |
| Identification | 1a | Identify the report as a protocol of a systematic review  Effectiveness and safety of photobiomodulation therapy in diabetic peripheral neuropathy: protocol for a systematic review and meta-analysis |  |  | 1-3 |
| Update | 1b | If the protocol is for an update of a previous systematic review, identify as such |  |  |  |
| **Registration** | 2 | If registered, provide the name of the registry (e.g., PROSPERO) and registration number in the Abstract  Registration: PROSPERO (registration number: CRD42023466586). |  |  | 38-39 |
| **Authors** | | | | | |
| Contact | 3a | Provide name, institutional affiliation, and e-mail address of all protocol authors; provide physical mailing address of corresponding author  Xuechun Fan ^1^, Guanchi Yan^2^, Jingsi Cao^1^, Yunyun Zhao^2^, Ying Wang ^1,^ Xiuge Wang ^2*^, Jia Mi ^2*^  1 College of Traditional Chinese Medicine, Changchun University of Chinese Medicine, Changchun, China  2 Department of Endocrinology, First Affiliated Hospital to Changchun University of Chinese Medicine, Changchun, China  Xuechun Fan 2445564126@qq.com; Guanchi Yan 372198682@qq.com; Jingsi Cao 1451440195@qq.com; Yunyun Zhao yephely0204@163.com; Ying Wang 486421187@qq.com  * Correspondence author: E-mail: xiuge_w@163.com (XW) E-mail: mijia8201@126.com (JM) |  |  | 4-12 |
| Contributions | 3b | Describe contributions of protocol authors and identify the guarantor of the review  Author Contributions  XF participated in the manuscript's writing, review, and editing. XF also played a role in conceptualizing the study, developing the methodology, and curating the data.  GY participated in the manuscript's writing, review, and editing. He also played a role in conceptualizing the study, and developing the methodology.  JC contributed to the reviewing, conceptualizing, and developing methodology for writing.  YZ contributed to the writing, review, conceptualizing, and software.  YW contributed to the writing, review, conceptualizing, and software.  XW participated in the manuscript's writing, review, and editing. She also played a role in conceptualizing the study and developing the methodology.  JM contributed to the writing by reviewing and editing the content. Additionally, JM played a role in conceptualizing and developing the methodology. |  |  | 242-254 |
| **Amendments** | 4 | If the protocol represents an amendment of a previously completed or published protocol, identify as such and list changes; otherwise, state plan for documenting important protocol amendments |  |  |  |
| **Support** | | | | | |
| Sources | 5a | Indicate sources of financial or other support for the review  Funding  Funding for this project was provided by the National Natural Science Foundation of China (No: 82205039), the Innovation Team and Talents Cultivation Program of National Administration of Traditional Chinese Medicine (No: ZYYCXTDD-202001), the Jilin Scientific and Technological Development Program (No: 20200201305JC), the National Key Research and Development Program of China (No: 2019YFC1709904) and Jilin Province Traditional Chinese Medicine Science and Technology Project (No: 2024021). |  |  | 255-262 |
| Sponsor | 5b | Provide name for the review funder and/or sponsor |  |  |  |
| Role of sponsor/funder | 5c | Describe roles of funder(s), sponsor(s), and/or institution(s), if any, in developing the protocol |  |  |  |
| **INTRODUCTION** | | | | | |
| **Rationale** | 6 | Describe the rationale for the review in the context of what is already known  Introduction: Diabetic peripheral neuropathy (DPN), a widely prevalent complication in patients with type 2 diabetes, exerts a significant influence on patients' overall health and financial circumstances. Photobiomodulation therapy is one of the means of physical therapy for DPN. Although preliminary findings suggest the efficacy of photobiomodulation therapy in alleviating peripheral neuropathy, the existing literature lacks substantial evidence regarding its safety and effectiveness specifically in the context of diabetes-related peripheral neuropathy. Therefore, we plan to arrive at more distinct findings through systematic evaluation and meta-analysis. |  |  | 16-23 |
| **Objectives** | 7 | Provide an explicit statement of the question(s) the review will address with reference to participants, interventions, comparators, and outcomes (PICO)  Classification of participants  Our study will include adults (over 18 years of age) with any form of diabetes who have been diagnosed with DPN. There will be no restrictions on the age, gender, ethnic background, or nationality of the participants who are enrolled. Exclusion will occur for studies involving pregnant or other types of peripheral neuropathy.  Type of outcome  The primary outcomes will include change of symptom scores (Neurological Impairment Scale [NIS] or similar scales), change of nerve conduction velocity. In addition to photobiomodulation therapy-related adverse events, the secondary outcomes will encompass quality of life (Health-Related Quality of Life scale or related scores), Change in pain (Visual Analogue Scale, Numeric Rating Scale and so on), fasting blood glucose, blood glucose 2 hours after eating, and glycosylated hemoglobin.  Type of interventions  There will be no restrictions on the type or frequency of light, the wavelength or equipment that produces light. The light can come from LEDs, low level lasers, or lamps. We will include randomized controlled trials that meet any of the following intervention comparisons: photobiomodulation therapy and pseudo photobiomodulation therapy, photobiomodulation therapy and placebo, photobiomodulation therapy and non-specific treatment other than conventional treatment, or photobiomodulation therapy plus another treatment and another treatment in addition to conventional treatment. |  |  | 98-118 |
| **METHODS** | | | | | |
| **Eligibility criteria** | 8 | Specify the study characteristics (e.g., PICO, study design, setting, time frame) and report characteristics (e.g., years considered, language, publication status) to be used as criteria for eligibility for the review  Types of study  We will include randomized controlled trials (RCTs) of photobiomodulation therapy for patients with diabetes peripheral neuropathy, while quasi-RCTs will not be specifically excluded. Non-randomized clinical trials, duplicated publications, conference records, reviews, meta-analyses, newspapers, guides, letters, other documents, and studies without full text will be omitted. Publication will be limited to English and Chinese, with no limitations on the date of publication.  Classification of participants  Our study will include adults (over 18 years of age) with any form of diabetes who have been diagnosed with DPN. There will be no restrictions on the age, gender, ethnic background, or nationality of the participants who are enrolled. Exclusion will occur for studies involving pregnant or other types of peripheral neuropathy.  Type of outcome  The primary outcomes will include change of symptom scores (Neurological Impairment Scale [NIS] or similar scales), change of nerve conduction velocity. In addition to photobiomodulation therapy-related adverse events, the secondary outcomes will encompass quality of life (Health-Related Quality of Life scale or related scores), Change in pain (Visual Analogue Scale, Numeric Rating Scale and so on), fasting blood glucose, blood glucose 2 hours after eating, and glycosylated hemoglobin.  Type of interventions  There will be no restrictions on the type or frequency of light, the wavelength or equipment that produces light. The light can come from LEDs, low level lasers, or lamps. We will include randomized controlled trials that meet any of the following intervention comparisons: photobiomodulation therapy and pseudo photobiomodulation therapy, photobiomodulation therapy and placebo, photobiomodulation therapy and non-specific treatment other than conventional treatment, or photobiomodulation therapy plus another treatment and another treatment in addition to conventional treatment.  Electronic searches  The study will encompass papers published from the inception until October 1, 2023, employing terminology such as photobiomodulation therapy, diabetic peripheral neuropathy, and randomized controlled trial. |  |  | 91-118  122-125 |
| **Information sources** | 9 | Describe all intended information sources (e.g., electronic databases, contact with study authors, trial registers, or other grey literature sources) with planned dates of coverage  Electronic searches  The study will encompass papers published from the inception until October 1, 2023, employing terminology such as photobiomodulation therapy, diabetic peripheral neuropathy, and randomized controlled trial. Web of Science, Embase, Cochrane Library, PubMed, AMED, Wanfang database, VIP database, China National Knowledge Infrastructure, and the Chinese Biomedical Literature database will all be queried during the search. Table 1 displays the detailed search method for PubMed.  Other search strategy  We will examine additional ongoing and unpublished studies listed in the WHO International Clinical Trial Registration Platform(https://trialsearch.who.int/), China Clinical Trial Registration Platform(http://www.chictr.org.cn/), and Clinical Trials.gov (https://clinicaltrials.gov/). Additionally, the relevant systematic reviews' reference lists will be thoroughly examined by manual means. The retrieval of gray literature conducted through the utilization of Google Scholar and opengrey.edu.  Missing data  In case the included studies are missing crucial information, we will reach out to the primary investigator to acquire the necessary details. If the necessary information cannot be obtained, we will exclude these studies. |  |  | 122-136  168-171 |
| **Search strategy** | 10 | Present draft of search strategy to be used for at least one electronic database, including planned limits, such that it could be repeated  Electronic searches  The study will encompass papers published from the inception until October 1, 2023, employing terminology such as photobiomodulation therapy, diabetic peripheral neuropathy, and randomized controlled trial. Web of Science, Embase, Cochrane Library, PubMed, AMED, Wanfang database, VIP database, China National Knowledge Infrastructure, and the Chinese Biomedical Literature database will all be queried during the search. Table 1 displays the detailed search method for PubMed.  Table 1. Search strategy in PubMed |  |  | 122-129 |
| ***STUDY RECORDS*** | | | | | |
| Data management | 11a | Describe the mechanism(s) that will be used to manage records and data throughout the review  Summary of findings  The GRADE will be employed to import data from Review Manager 5.4, in order to generate a table presenting the summary of findings[25]. Two researchers will independently assess the quality of evidence. Every result will be categorized into one of four ratings: high, medium, low, or very low. |  |  | 202-206 |
| Selection process | 11b | State the process that will be used for selecting studies (e.g., two independent reviewers) through each phase of the review (i.e., screening, eligibility, and inclusion in meta-analysis)  Study selection  Two researchers will conduct separate evaluations of the studies. The outcomes will be examined and any discrepancies will be discussed. During the course of the research period, any disagreements will be effectively solved by engaging in discussions or negotiations with a third researcher. Figure 1 displays a flowchart depicting the screening process, which is derived from the PRISMA flow diagram 2020[23]. |  |  | 137-143 |
| Data collection process | 11c | Describe planned method of extracting data from reports (e.g., piloting forms, done independently, in duplicate), any processes for obtaining and confirming data from investigators  Data collection and analysis  After reading the complete text of the chosen papers, two researchers will independently extract the information:  1. The papers contain essential details (the name of primary author, the year of publication, country, language, and race).  2. Key characteristics of the patients (sample size, inclusion criteria, effectiveness criteria, Diabetes duration/DPN duration, and demographic baseline).  3. The details regarding the intervention and control groups, including the duration of intervention, type of intervention, and frequency of intervention.  4. Methodological characteristics (blinding and hidden allocation sequence).  5. The results include primary outcomes (symptom score, nerve conduction velocity) and secondary outcomes (quality of life scores, 2-h postprandial blood glucose, fasting blood glucose, glycosylated hemoglobin, and any adverse events associated with photobiomodulation therapy).  All conflicts will be settled by participating in discourse or deliberation with a tertiary researcher. |  |  | 144-159 |
| **Data items** | 12 | List and define all variables for which data will be sought (e.g., PICO items, funding sources), any pre-planned data assumptions and simplifications  Types of study  We will include randomized controlled trials (RCTs) of photobiomodulation therapy for patients with diabetes peripheral neuropathy, while quasi-RCTs will not be specifically excluded. Non-randomized clinical trials, duplicated publications, conference records, reviews, meta-analyses, newspapers, guides, letters, other documents, and studies without full text will be omitted. Publication will be limited to English and Chinese, with no limitations on the date of publication.  Classification of participants  Our study will include adults (over 18 years of age) with any form of diabetes who have been diagnosed with DPN. There will be no restrictions on the age, gender, ethnic background, or nationality of the participants who are enrolled. Exclusion will occur for studies involving pregnant or other types of peripheral neuropathy.  Type of outcome  The primary outcomes will include change of symptom scores (Neurological Impairment Scale [NIS] or similar scales), change of nerve conduction velocity. In addition to photobiomodulation therapy-related adverse events, the secondary outcomes will encompass quality of life (Health-Related Quality of Life scale or related scores), Change in pain (Visual Analogue Scale, Numeric Rating Scale and so on), fasting blood glucose, blood glucose 2 hours after eating, and glycosylated hemoglobin.  Type of interventions  There will be no restrictions on the type or frequency of light, the wavelength or equipment that produces light. The light can come from LEDs, low level lasers, or lamps. We will include randomized controlled trials that meet any of the following intervention comparisons: photobiomodulation therapy and pseudo photobiomodulation therapy, photobiomodulation therapy and placebo, photobiomodulation therapy and non-specific treatment other than conventional treatment, or photobiomodulation therapy plus another treatment and another treatment in addition to conventional treatment.  Data collection and analysis  After reading the complete text of the chosen papers, two researchers will independently extract the information:  1. The papers contain essential details (the name of primary author, the year of publication, country, language, and race).  2. Key characteristics of the patients (sample size, inclusion criteria, effectiveness criteria, Diabetes duration/DPN duration, and demographic baseline).  3. The details regarding the intervention and control groups, including the duration of intervention, type of intervention, and frequency of intervention.  4. Methodological characteristics (blinding and hidden allocation sequence).  5. The results include primary outcomes (symptom score, nerve conduction velocity) and secondary outcomes (quality of life scores, 2-h postprandial blood glucose, fasting blood glucose, glycosylated hemoglobin, and any adverse events associated with photobiomodulation therapy).  All conflicts will be settled by participating in discourse or deliberation with a tertiary researcher.  Funding  Funding for this project was provided by the National Natural Science Foundation of China (No: 82205039), the Innovation Team and Talents Cultivation Program of National Administration of Traditional Chinese Medicine (No: ZYYCXTDD-20200c1), the Jilin Scientific and Technological Development Program (No: 20200201305JC), the National Key Research and Development Program of China (No: 2019YFC1709904) and Jilin Province Traditional Chinese Medicine Science and Technology Project (No: 2024021). |  |  | 91-118  144-159  255-262 |
| **Outcomes and prioritization** | 13 | List and define all outcomes for which data will be sought, including prioritization of main and additional outcomes, with rationale  Type of outcome  The primary outcomes will include change of symptom scores (Neurological Impairment Scale [NIS] or similar scales), change of nerve conduction velocity. In addition to photobiomodulation therapy-related adverse events, the secondary outcomes will encompass quality of life (Health-Related Quality of Life scale or related scores), Change in pain (Visual Analogue Scale, Numeric Rating Scale and so on), fasting blood glucose, blood glucose 2 hours after eating, and glycosylated hemoglobin. |  |  | 103-109 |
| **Risk of bias in individual studies** | 14 | Describe anticipated methods for assessing risk of bias of individual studies, including whether this will be done at the outcome or study level, or both; state how this information will be used in data synthesis  Risk of bias assessment  The Cochrane Risk of Bias 2 (RoB 2) tool[22] and Physiotherapy Evidence Database (PEDro) scale(https://www.pedro.org.au/english/downloads/pedro-scale/) were employed, two researchers will individually evaluate the potential for bias. The evaluation and assessment of all the studies will be conducted based on participant blinding, allocation concealment, outcome assessment blinding, result data integrity, selective outcome reporting, along with potential biases. The assessment results will be classified as low, high, or uncertain risk.  Data synthesis  RevMan V.5.4 software will be used for meta-analysis. The weighted mean difference of the 95% CI will be used for continuous data and the risk ratios of the 95% CI will be used for dichotomous data. In case there are less than or equal to 3 studies included, we will furnish a descriptive and qualitative overview.  Subgroup analysis  Analysis of subgroups will be carried out, taking into account the following factors: Photobiomodulation therapy type, diabetes type, intervention duration, course of diabetes, and duration of DPN.  Sensitivity analysis  To measure the resilience and dependability of the findings, a sensitivity analysis will be conducted. Studies with a significant bias will be excluded. Furthermore, the impact of chosen models will be taken into account. Step-wise rejection method is used for sensitivity analysis.  Meta-regression analysis  The process of meta-regression will be carried out in a same manner to linear regression, where the study estimate will serve as the dependent variable and the study characteristics[24] will act as the independent variables. In addition, we will use R language for meta-regression analysis. |  |  | 160-167  183-201 |
| ***DATA*** | | | | | |
| **Synthesis** | 15a | Describe criteria under which study data will be quantitatively synthesized  Evaluation of reporting biases  Funnel plots can be utilized to reveal biases of reports when the number of included studies exceeds ten.  Data synthesis  RevMan V.5.4 software will be used for meta-analysis. The weighted mean difference of the 95% CI will be used for continuous data and the risk ratios of the 95% CI will be used for dichotomous data. In case there are less than or equal to 3 studies included, we will furnish a descriptive and qualitative overview. |  |  | 180-187 |
|  | 15b | If data are appropriate for quantitative synthesis, describe planned summary measures, methods of handling data, and methods of combining data from studies, including any planned exploration of consistency (e.g., *I* ^2^, Kendall’s tau)  Evaluation of heterogeneity  To evaluate the heterogeneity of the studies incorporated, we will employ the χ2 test and I2 value [22]. When the P values in the χ2 test are greater than 0.05 or the I2 is less than 50%, it indicates that there is homogeneity among the studies. When the value of P is less than or equal to 0.05 and the value of I2 is greater than or equal to 50%, this finding suggests that there is variability or diversity among the studies. To analyze the heterogeneity, we will implement subgroup analysis and meta-regression to analyze the origin of variability.  Data synthesis  RevMan V.5.4 software will be used for meta-analysis. The weighted mean difference of the 95% CI will be used for continuous data and the risk ratios of the 95% CI will be used for dichotomous data. In case there are less than or equal to 3 studies included, we will furnish a descriptive and qualitative overview. |  |  | 172-179  183-187 |
|  | 15c | Describe any proposed additional analyses (e.g., sensitivity or subgroup analyses, meta-regression)  Subgroup analysis  Analysis of subgroups will be carried out, taking into account the following factors: Photobiomodulation therapy type, diabetes type, intervention duration, course of diabetes, and duration of DPN.  Sensitivity analysis  To measure the resilience and dependability of the findings, a sensitivity analysis will be conducted. Studies with a significant bias will be excluded. Furthermore, the impact of chosen models will be taken into account. Step-wise rejection method is used for sensitivity analysis.  Meta-regression analysis  The process of meta-regression will be carried out in a same manner to linear regression, where the study estimate will serve as the dependent variable and the study characteristics[24] will act as the independent variables. In addition, we will use R language for meta-regression analysis. |  |  | 188-201 |
|  | 15d | If quantitative synthesis is not appropriate, describe the type of summary planned  Data synthesis  RevMan V.5.4 software will be used for meta-analysis. The weighted mean difference of the 95% CI will be used for continuous data and the risk ratios of the 95% CI will be used for dichotomous data. In case there are less than or equal to 3 studies included, we will furnish a descriptive and qualitative overview.  Summary of findings  The GRADE will be employed to import data from Review Manager 5.4, in order to generate a table presenting the summary of findings[25]. Two researchers will independently assess the quality of evidence. Every result will be categorized into one of four ratings: high, medium, low, or very low. |  |  | 183-187  202-206 |
| **Meta-bias(es)** | 16 | Specify any planned assessment of meta-bias(es) (e.g., publication bias across studies, selective reporting within studies)  Risk of bias assessment  The Cochrane Risk of Bias 2 (RoB 2) tool[22] and Physiotherapy Evidence Database (PEDro) scale(https://www.pedro.org.au/english/downloads/pedro-scale/) were employed, two researchers will individually evaluate the potential for bias. The evaluation and assessment of all the studies will be conducted based on participant blinding, allocation concealment, outcome assessment blinding, result data integrity, selective outcome reporting, along with potential biases. The assessment results will be classified as low, high, or uncertain risk.  Missing data  In case the included studies are missing crucial information, we will reach out to the primary investigator to acquire the necessary details. If the necessary information cannot be obtained, we will exclude these studies.  Evaluation of heterogeneity  To evaluate the heterogeneity of the studies incorporated, we will employ the χ2 test and I2 value [22]. When the P values in the χ2 test are greater than 0.05 or the I2 is less than 50%, it indicates that there is homogeneity among the studies. When the value of P is less than or equal to 0.05 and the value of I2 is greater than or equal to 50%, this finding suggests that there is variability or diversity among the studies. To analyze the heterogeneity, we will implement subgroup analysis and meta-regression to analyze the origin of variability.  Evaluation of reporting biases  Funnel plots can be utilized to reveal biases of reports when the number of included studies exceeds ten.  Subgroup analysis  Analysis of subgroups will be carried out, taking into account the following factors: Photobiomodulation therapy type, diabetes type, intervention duration, course of diabetes, and duration of DPN.  Sensitivity analysis  To measure the resilience and dependability of the findings, a sensitivity analysis will be conducted. Studies with a significant bias will be excluded. Furthermore, the impact of chosen models will be taken into account. Step-wise rejection method is used for sensitivity analysis.  Meta-regression analysis  The process of meta-regression will be carried out in a same manner to linear regression, where the study estimate will serve as the dependent variable and the study characteristics[24] will act as the independent variables. In addition, we will use R language for meta-regression analysis. |  |  | 160-182  188-201 |
| **Confidence in cumulative evidence** | 17 | Describe how the strength of the body of evidence will be assessed (e.g., GRADE)  Summary of findings  The GRADE will be employed to import data from Review Manager 5.4, in order to generate a table presenting the summary of findings[25]. Two researchers will independently assess the quality of evidence. Every result will be categorized into one of four ratings: high, medium, low, or very low. |  |  | 202-206 |
