# Supplementary material for: Effectiveness and safety of photobiomodulation therapy in diabetic peripheral neuropathy: Protocol for a systematic review and meta-analysis
Source: PLoS One. 2024 Aug 26;19(8):e0308537. doi: 10.1371/journal.pone.0308537 (PMC11346721; doi:10.1371/journal.pone.0308537)
Supplement: S1 Table — (DOCX) [file pone.0308537.s002.docx]

Pubmed

" Photobiomodulation Therapy "[Mesh] OR

所有字段：((((((((((((((((((((((Phototherapy) OR (Phototherapies)) OR (Photoradiation therapy)) OR (Therapy, Photoradiation)) OR (Photoradiation Therapies)) OR (Therapies, Photoradiation)) OR (Light Therapy)) OR (Light Therapies)) OR (Therapies, Light)) OR (Therapy, Light)) OR (Light emitting diode therapy)) OR (Low level light therapy)) OR (Light)) OR (photobiomodulation)) OR (Laser Phototherapy)) OR (infrared)) OR (Low level laser therapy)) OR (Cold laser)) OR (red light)) OR (photon)) OR (ultraviolet)) OR (optical energy)) OR (irradiation)) OR (super lizer)) OR (photon stimulation)) OR (monochromatic infrared photoenergy)) OR (monochromatic infrared phototherapy)

**AND**

"Diabetic Neuropathies"[Mesh] OR

所有字段：(((((((((((((((((((((((((((((((((((((((((((((Diabetic Neuropathy) OR (Neuropathies, Diabetic)) OR (Neuropathy, Diabetic)) OR (Diabetic Autonomic Neuropathy)) OR (Autonomic Neuropathies, Diabetic)) OR (Autonomic Neuropathy, Diabetic)) OR (Diabetic Autonomic Neuropathies)) OR (Neuropathies, Diabetic Autonomic)) OR (Neuropathy, Diabetic Autonomic)) OR (Diabetic Neuralgia)) OR (Diabetic Neuralgias)) OR (Neuralgias, Diabetic)) OR (Diabetic Neuropathy, Painful)) OR (Diabetic Neuropathies, Painful)) OR (Neuropathies, Painful Diabetic)) OR (Neuropathy, Painful Diabetic)) OR (Painful Diabetic Neuropathies)) OR (Painful Diabetic Neuropathy)) OR (Neuralgia, Diabetic)) OR (Symmetric Diabetic Proximal Motor Neuropathy)) OR (Asymmetric Diabetic Proximal Motor Neuropathy)) OR (Diabetic Asymmetric Polyneuropathy)) OR (Asymmetric Polyneuropathies, Diabetic)) OR (Asymmetric Polyneuropathy, Diabetic)) OR (Diabetic Asymmetric Polyneuropathies)) OR (Polyneuropathies, Diabetic Asymmetric)) OR (Polyneuropathy, Diabetic Asymmetric)) OR (Diabetic Mononeuropathy)) OR (Diabetic Mononeuropathies)) OR (Mononeuropathies, Diabetic)) OR (Mononeuropathy, Diabetic)) OR (Diabetic Mononeuropathy Simplex)) OR (Diabetic Mononeuropathy Simplices)) OR (Mononeuropathy Simplex, Diabetic)) OR (Mononeuropathy Simplices, Diabetic)) OR (Simplex, Diabetic Mononeuropathy)) OR (Simplices, Diabetic Mononeuropathy)) OR (Diabetic Amyotrophy)) OR (Amyotrophies, Diabetic)) OR (Amyotrophy, Diabetic)) OR (Diabetic Amyotrophies)) OR (Diabetic Polyneuropathy)) OR (Diabetic Polyneuropathies)) OR (Polyneuropathies, Diabetic)) OR (Polyneuropathy, Diabetic)) OR (Diabetic peripheral neuropathy)

**AND**

"Randomized Controlled Trial" [Publication Type] OR

所有字段：((((((((((Controlled clinical trial) OR (Clinical Trial)) OR (Randomised)) OR (Randomly)) OR (Trial)) OR (Random allocation)) OR (Clinical Study)) OR (Randomized Controlled Trial)) OR (Controlled)) OR (Effect)) OR (application)

**NOT**

所有字段：(animals) OR (cell)

China National Knowledge Infrastructure

SU=光+光疗+激光+光生物调节+红光+红外+光子+紫外+红外线+光能+照射+直线偏光红外线+紫外线+光刺激+单色红外光能+单色红外光疗

AND

SU=糖尿病周围神经病+糖尿病周围神经病变+消渴痹证+消渴病痹症+消渴痹症+周痹

AND

SU=随机对照试验+随机+对照+试验+临床疗效观察+疗效+临床+治疗+影响+应用

NOT

SU=动物+细胞
